# Perioperative Prevention of Venous Thromboembolism in Abdominal Surgery Patients Based on the Caprini or the Padua Risk Score—A Single Centre Prospective Observational Study

**DOI:** 10.3390/life12111843

**Published:** 2022-11-11

**Authors:** Jasna Klen, Gašper Horvat, Aleš Blinc

**Affiliations:** 1 Department of Abdominal Surgery, University Medical Centre Ljubljana, 1525 Ljubljana, Slovenia; 2 Faculty of Medicine, University of Ljubljana, 1000 Ljubljana, Slovenia; 3 Department of Vascular Diseases, University Medical Centre Ljubljana, 1525 Ljubljana, Slovenia

**Keywords:** abdominal surgery, elective, venous thromboembolism, Caprini score, Padua score, thromboprophylaxis, postoperative bleeding

## Abstract

Surgical patients should receive perioperative thromboprophylaxis based on risk assessment, and the Caprini score is validated for this purpose. Whether the Padua score, originally devised for medical patients, can be useful in surgical patients remains to be fully clarified. This study aimed to evaluate perioperative thromboprophylaxis based on the Caprini or the Padua score in elective abdominal surgery. A total of 223 patients undergoing elective abdominal surgery for malignant or benign disease were prospectively evaluated. The patients were divided into two groups in which thromboprophylaxis was prescribed according to either the Caprini score (n = 122) or the Padua score (n = 101). Patients with high-risk scores in both groups received nadroparin. The alternate risk score in each group was calculated for evaluation purposes only. During a 3-month follow-up, we assessed patients for symptomatic venous thromboembolism (VTE), bleeding, or mortality. In the Caprini score group, 87 patients (71%) had a high risk for VTE (≥5 points), while 38 patients (38%) had a high risk for VTE (≥4 points) in the Padua score group; *p* < 0.00001. The overall correlation between the Caprini and Padua scores was moderate (r= 0.619), with 85 patients having high Caprini and discordant Padua scores. Ten patients died during follow-up (4.5%), and five developed non-fatal symptomatic VTE (2.2%). Among the five major bleeding incidents recorded (1.8%), two cases were possibly associated with pharmacological thromboprophylaxis. The incidence of adverse outcomes did not differ between the two groups. The odds ratio for adverse outcomes was significantly higher with a high Caprini or Padua risk score, malignant disease, age ≥65 years, and active smoking. We found no significant differences in adverse outcomes between abdominal surgical patients who received perioperative thromboprophylaxis based on either the Caprini or the Padua risk score. However, a discordant Padua score was noted in almost 40% of patients who had a high Caprini score, suggesting that the latter may be more sensitive than the Padua score in surgical patients.

## 1. Introduction

Venous thromboembolism (VTE), which comprises deep vein thrombosis (DVT) and pulmonary embolism (PE), is a potential complication that significantly contributes to morbidity and mortality during the perioperative and postoperative periods [1]. Patients undergoing abdominal surgical procedures for benign or malignant pathologies are at increased risk for VTE. In general surgical patients with low preoperative risk for VTE, the incidence of symptomatic VTE is 0.4% [2]. Several factors contribute to the increased risk of VTE in surgical patients, including immobility and venous stasis after surgery, vascular endothelial injury, platelet activation, overexpression of procoagulants in cancer patients, chemotherapy, tissue growth factor overexpression in monocytes, etc. [3]. In addition, there are also the patient’s personal risk factors for VTE such as inherited or acquired thrombophilia, increased body mass index, advanced age, smoking, oral contraceptives use, previous VTE, etc. [4]. Infection with SARS-CoV-2 is nowadays also considered a risk factor for VTE [5,6,7].

Several modalities of thromboprophylaxis are used in the early and later postoperative periods to prevent VTE in surgical patients. It is now generally accepted that all cancer patients undergoing abdominal or pelvic surgery should receive pharmacological thromboprophylaxis in the form of low-molecular-weight heparin or unfractionated heparin for up to 28 days post-surgery [8,9,10,11]. Mechanical thromboprophylaxis, such as intermittent pneumatic compression and compressive socks, can be used instead of pharmacological thromboprophylaxis in low-risk patients or as an additional strategy in high-risk patients [10,12]. Mechanical thromboprophylaxis has proven beneficial when used until patient mobilisation or for the duration of the hospitalisation [9,12].

Different risk scores are used to identify patients with an increased risk of VTE. The Caprini score is an internationally recognised and validated clinical tool devised to help recognise patients with a higher risk for VTE after surgery [10,13,14,15,16,17]. The Caprini score categorises patients into five different risk groups for VTE: very low risk (0 points), low risk (1–2 points), moderate risk (3–4 points), high risk (5–9 points), and very high risk (>9 points) [17]. Different mechanical and pharmacological prophylaxis, or a combination of both, is recommended depending on the risk group. A score of 5 points represents the cut-off value at which patients are deemed at high risk and recommended to receive pharmacological thromboprophylaxis with or without mechanical thromboprophylaxis. In patients with low or moderate risk, certain types of mechanical thromboprophylaxis are recommended. Patients that score 0 points are advised only early ambulation.

The Padua score is a validated clinical tool devised to recognise medical patients at high or low risk for VTE [18,19]. The score categorises patients based on different parameters into two VTE risk groups: a high-risk group for whom pharmacological thromboprophylaxis is indicated, and a lower-risk group for whom medical prophylaxis is not indicated, but mechanical prophylaxis may be considered. The score ranges from 0 points, the lowest possible risk score, to 20 points, the maximal possible score. A score of 4 points or more is considered high-risk [18].

The Padua score has not been routinely used for surgical patients, but has recently been tested in a tertiary institution, and its power to predict DVT in surgical patients has been found to be only moderately lower than that of the Caprini score [20]. An important disadvantage of the Caprini score is the myriad of parameters needed to be evaluated, while the Padua score is calculated more easily (Supplementary Material Tables S1 and S2). Using a structured approach to prevention of VTE is preferable over arbitrary clinical decisions [2].

We performed a prospective observational study to evaluate the occurrence of symptomatic VTE in elective abdominal surgery patients in whom the mode of thromboprophylaxis was based on the Caprini or the Padua risk score, with the other score also being calculated. Our aim was to test for possible discrepancies in identifying high-risk patients and to compare the incidence of symptomatic VTE. We also evaluated the bleeding risk associated with pharmacological thromboprophylaxis.

## 2. Patients and Methods

We prospectively recruited 227 patients scheduled for elective abdominal surgery for either benign or malignant pathology in the period from February 2020 to July 2021, who were not already on anticoagulation treatment for other indications such as mechanical heart valve, cardiovascular disease, previous VTE, etc. The benign indications for surgery in the recruited patients included gallstones or polyps of the gallbladder, different types of abdominal hernia, planned occlusion of an ileostomy, intestinal stenosis due to inflammatory bowel disease, rectal prolapse, anal fissures, and fistulas (Supplementary Material Table S1). Among malignant pathologies, the most common were adenocarcinoma or other types of malignant tumours of the colon, pancreas, stomach, or liver (Supplementary Material, Table S2). For the final analysis, patients had to complete a 3-month postoperative follow-up. All patients provided written consent before inclusion, and all study protocols were reviewed and approved by the National Medical Ethics Committee of the Republic of Slovenia (nr. 0120-90/2020-6).

A thorough medical history was obtained from all patients, following which physical examination and routine laboratory tests were performed. The risk of VTE was initially assessed by either the Caprini or the Padua score according to the attending physician’s discretion, and the obtained score was used for deciding on VTE prophylaxis. The alternate risk score was then calculated for evaluation purposes only. The Caprini and Padua score questionnaires are provided in the Supplementary Material (Tables S1 and S2).

Patients with a high risk for VTE, indicated by ≥5 points with the Caprini score or ≥4 points with the Padua score, were prescribed pharmacological thromboprophylaxis with nadroparin, a low-molecular-weight heparin (LMWH). Patients with body weight ≤80 kg received 3800 I.U. daily, and those ≥80 kg received 5700 I.U. daily. Patients who were operated on because of benign pathology received nadroparin for the duration of their hospital stay, ranging from 3–10 days. On the other hand, patients with cancer received extended postoperative thromboprophylaxis, with nadroparin self-administered after hospital discharge to the 28th postoperative day. The first dose of LMWH was applied the night before the operation, and the first postoperative dose was administered approximately 12 h after surgery. The subsequent doses were administered in daily intervals. None of the patients who received pharmacological thromboprophylaxis had contraindications such as glomerular filtration rate under 30 mL/min, thrombocytopenia, pregnancy, or allergies [21]. Mechanical thromboprophylaxis with compression stockings or intermittent pneumatic compression was added to pharmacological thromboprophylaxis or used alone at the discretion of the attending physician.

Patients with low or intermediate VTE risk, i.e., 1–4 points with the Caprini score or 1–3 points with the Padua score, received only mechanical thromboprophylaxis: calf compression stockings that patients wore until mobilisation, or intermittent pneumatic compression that was applied until patient mobilisation.

After discharge from the hospital, the patients were followed up for 3 months, and the occurrence of symptomatic VTE, bleeding, or death was assessed. Follow-up was performed in the ambulatory setting during regular postoperative check-ups and by telephone. Death was confirmed by the hospital or electronic healthcare records. Patients were interviewed about clinical symptoms of VTE: swelling or pain in the lower or upper limbs, dyspnoea, tachycardia, chest pain, loss of consciousness [22,23]. In case of VTE suspicion, venous ultrasound examination or pulmonary CT angiography was performed accordingly. Our decision not to perform venous ultrasound screening in all patients was based on its low sensitivity for calf venous thrombosis, although realizing that asymptomatic venous thrombosis might become clinically relevant [24], Since infection by SARS-CoV-2 contributes to VTE risk, we also recorded PCR-confirmed cases of COVID-19 in the postoperative period. 

Bleeding was evaluated by clinical, laboratory, or radiological signs in the early postoperative period. Major bleeding was defined according to the International Society on Thrombosis and Haemostasis (ISTH) criteria [25]. 

## 3. Statistical Analysis

The mean and standard deviation (SD) were used to describe the continuous variables whose distribution did not differ significantly from normal according to the Kolmogorov–Smirnov test. The median and 25–75% range were used to describe continuous variables with a non-normal distribution. Frequencies were used to describe categorical variables. The Pearson correlation between the Caprini and Padua scores was calculated. For comparison of groups with approximately normally distributed continuous variables, the two-tailed Student’s t-test was used. The Mann–Whitney U test was used to compare groups of non-normally distributed continuous variables, while the Chi-squared test was used to compare the distribution of categorical variables. All tests were two-sided, and the significance level was set to 0.05. The odds ratio (OD) with a 95% confidence interval (CI) for death or VTE was calculated for various sub-groups. The tests were performed with Microsoft Excel or online calculators (https://www.socscistatistics.com and www.gigacalculator.com/calculators/odds-ratio-calculator.php (accessed on 27 September 2022)).

## 4. Results

Of the 227 recruited patients, 223 were included in the final analysis, while 4 were excluded due to incomplete follow-up. The general baseline characteristics of the patients and the prevalence of confirmed COVID-19 in the 3-month observation period are presented in Table 1. The table also describes and compares the characteristics of patients in the Caprini or the Padua score groups. Although the two groups were not randomized, they did not differ in baseline characteristics.

Among 122 patients in whom the Caprini score was used for prescribing thromboprophylaxis, 89 high- and very-high-risk patients received pharmacologic thromboprophylaxis with LMWH; pneumatic compression was used in 23 patients, and a combination of both methods in 15 patients. Among 101 patients in whom the Padua score was used for prescribing thromboprophylaxis, LMWH was used in 43 patients, pneumatic compression in 3 patients and a combination of both methods in 1 patient. In the Padua score group, significantly smaller proportions of patients were prescribed LMWH, pneumatic compression or a combination of both methods than in the Caprini group (*p* ≤ 0.001 for all three comparisons). Elastic stockings were prescribed in 96 patients in the Caprini group and in 63 patients in the Padua group, *p* < 0.001.

The distribution of the Caprini score values and the Padua score values, as well as the adverse outcomes, are listed in Table 2 for the whole cohort and for the subgroups where one or the other score was used for prescribing thromboprophylaxis. No significant differences were found. In the subgroup where the Caprini score was used for prescribing thromboprophylaxis, 87 out of 122 patients (71%) had a high risk for VTE (≥5 points), while in the Padua score subgroup, 38 out of 101 patients (38%) had a high risk for VTE (≥4 points), *p* < 0.00001.

**Figure 1:**
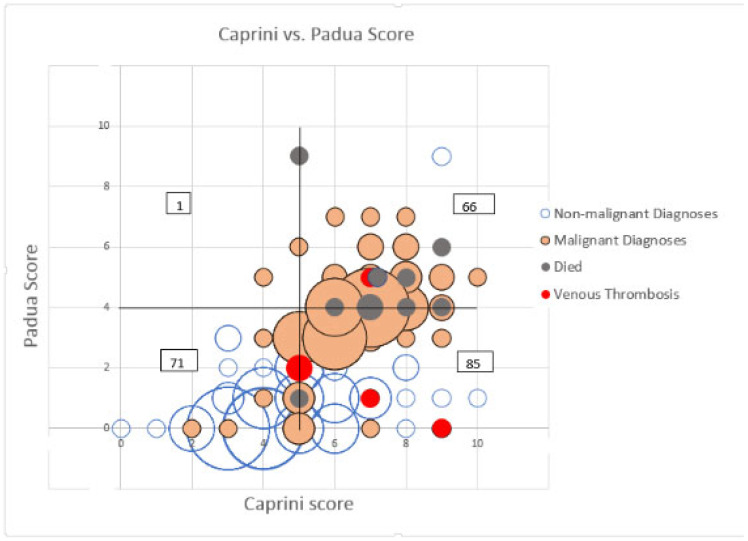
Bubble graph showing the distribution of Caprini and Padua score values for the patients, separated for subjects with malignant diagnoses (shaded bubbles) and subjects with benign pathology (empty bubbles). The grey circles denote the scores of the patients who died, while the red circles denote the scores of patients who developed deep venous thrombosis. The solid lines divide the non-high and high score values, while the numbers in rectangles show the number of subjects in each quadrant.

The graphical relations of the Caprini and Padua scores in our cohort are presented in Figure 1. Patients who were operated on because of malignant disease had higher values than patients with benign diagnoses on both scores: median Padua score 4 (3–4) vs. 0 (0–1) points, *p* < 0.00001; Caprini core 7 (6–7) vs. 4 (3–5.75) points, *p* < 0.00001.

The overall Pearson correlation between the Caprini and Padua scores was only moderate, albeit highly significant (r = 0.619, *p* < 0.00001). Notably, 85 out of 223 patients had a non-high Padua score (3 points) and, conversely, a high Caprini score (≥5 points), but only 1 out of the 223 patients had a non-high Caprini score (≤4 points) and a simultaneously high Padua score (≥4 points).

The odds of developing an adverse outcome, i.e., death or symptomatic VTE, are shown for different sub-groups in Table 3. All patients who died or developed symptomatic VTE had a high Caprini score. The odds ratio for death was also significantly higher with a high Padua score compared to a non-high Padua score. Patients with malignant diagnoses had a higher odds ratio for death than those with benign diagnoses, but the odds ratio for VTE did not differ among the two groups. Our cohort did not show an association between adverse outcomes and gender, but patients aged 65 years or older had a higher odds ratio for death and/or symptomatic VTE. In smokers, we detected a higher odds ratio only for the combined adverse outcome. We did not find an elevated odds ratio for adverse outcomes with COVID-19.

In our cohort, five patients suffered non-fatal VTE during the follow-up period. All five had confirmed DVT, and none had confirmed PE. Three patients were detected in the first postoperative month (incidence of 1.4%), and one patient each in the second (incidence of 0.5%) and third (incidence of 0.5%) postoperative months. The cumulative 3-month incidence of VTE in our cohort was thus 2.4%. Four cases occurred among patients with benign disease and only one among patients with malignant disease. The patient with malignancy was the only one in whom DVT occurred during prophylactic treatment with LMWH, while the other four patients suffered DVT while they were not or no longer on LMWH. Furthermore, the patient with malignancy was the only one among the symptomatic DVT patients with concordantly high Caprini and Padua risk scores; the other four patients had low Padua scores but high Caprini scores. Two out of the four patients with benign pathology and DVT received thromboprophylaxis according to the Caprini score, i.e., nadroparin for the duration of the hospital stay; while the other two patients with DVT did not receive pharmacological thromboprophylaxis, as they were treated in accordance with their non-high Padua scores. 

Among the 10 patients who died during the 3-month postoperative follow-up, nine had malignant disease. Of these, the cause of death was septic shock after surgery in four cases, postoperative haemorrhagic shock in one case, and progression of the underlying malignant disease in four cases. The only patient without malignancy who died was a 64-year-old woman with transplanted liver who suffered fatal postoperative septic shock.

We recorded 15 PCR-confirmed infections with SARS-CoV-2 during the observational period. One of the patients with COVID-19 died, and one developed DVT, but in our cohort, the odds ratio for adverse outcomes was not increased by COVID-19.

In five patients, non-fatal major bleeding occurred after surgery. Three cases were directly related to the surgical procedure, while two were probably related to pharmacological thromboprophylaxis. Thus, two out of 114 (1.7%) patients who received pharmacological thromboprophylaxis suffered a major bleed related to LMWH. The odds ratio for major bleeding with pharmacological thromboprophylaxis vs. no pharmacological thromboprophylaxis could not be calculated because there were no bleeds in the group that was not prescribed LMWH.

## 5. Discussion

Our single-centre, prospective observational study revealed a low incidence of VTE-related adverse events at 3-month follow-up among abdominal surgery patients who were prescribed perioperative thromboprophylaxis based on either the Caprini or the Padua risk score, with no significant difference in adverse outcome between both groups.

Our analysis showed a higher risk of developing VTE in the group of patients who achieved ≥5 points according to the Caprini score or ≥4 points with the Padua score. Panucci et al. investigated the benefits and risks of pharmacological thromboprophylaxis in patients from a wide range of surgical specialties who were evaluated with the Caprini score and noted that patients with a Caprini score of ≥7 had the highest risk for VTE but represented a minority of patients [13]. These findings are in agreement with our results. Our results further demonstrate that surgical patients with malignant pathology had higher values than those with benign diseases on both risk scores. However, in our study, 85 (approximately 40%) patients with a low Padua score had a high Caprini score. The Caprini risk score has already been proven reliable and highly accurate in identifying surgical patients with high VTE risk [14,15,26]. On the other hand, the Padua score is not routinely used for surgical patients and has not yet been validated in large randomised studies. Nevertheless, a few studies have compared the effectiveness of the Caprini and Padua risk scores. Liu et al. reported that the Caprini score more often classifies patients into high and very-high risk score groups, has higher sensitivity and positive and negative predictive values than the Padua score, and is, therefore, more effective in recognising patients at risk for VTE [27]. These findings were reaffirmed by Zhou et al. [28]. Among cancer patients undergoing chemotherapy, the Padua score identified fewer high-risk patients than the Caprini score [29]. The predictive value of the Caprini and Padua scores for patients with malignant pathology is comparable. On the other hand, the Caprini score has a higher predictive value for patients with benign pathology [30]. A recent study by Anand et al. compared the Caprini and Padua scores in surgical patients and concluded that even though both risk scores can be used to predict VTE in surgical patients, the Caprini score is superior [20]. A Chinese study that included surgical and medical patients found that 20% of patients with DVT had achieved more than 10 on the Caprini score [30].

Although the two risk score groups were not randomized, they did not differ significantly in age, gender distribution, proportion of malignant or non-malignant diagnoses, and smoking status. No difference in the incidence of symptomatic VTE was found between the groups, and the overall incidence of postoperative symptomatic VTE was 2.4%. This is in line with the low estimated risk for VTE after surgery for patients in the very-low and low-risk groups, the estimated 0.7% incidence in the moderate risk group and an incidence of 1.4% to 10.7% in the high and very high-risk groups, depending on individual risk factors [13,15,16,31].

We recorded no cases of symptomatic PE, which is not surprising given its low incidence even among surgical patients receiving no thromboprophylaxis (ranges between 0.2% and 4–10% for the highest-risk patients) [32]. For patients receiving mechanical thromboprophylaxis, the reported incidence of symptomatic PE ranges from 0.13% to 0.65% [8] and 0.56% [32] to 1.3% [33]. Interestingly, our incidence of symptomatic VTE was lower among oncological patients, but due to the low values, the difference was not statistically significant. We attribute the low incidence of VTE among cancer patients to extended postoperative thromboprophylaxis with nadroparin, as the efficacy of LMWH in preventing VTE in cancer surgical patients has been firmly established [8,11,34]. Due to the low incidence of VTE, no association was found in our cohort with age, gender, smoking status, or COVID-19, although these are well-known VTE risk factors [35,36]. The patients in our cohort with benign pathology in whom DVT occurred were not or no longer on LMWH at the time of diagnosis. Among our DVT patients with malignant pathology, only one simultaneously had a high Caprini and Padua risk score. Two out of four patients with benign diagnosis and DVT received thromboprophylaxis according to their Caprini score, while the other 2 patients with DVT did not receive pharmacological thromboprophylaxis due to a non-high Padua score. It might be argued that the wrong score was used in these two surgical patients. Inappropriate administration of pharmacological thromboprophylaxis according to the Caprini score has been reported in a large percentage of surgical patients [37]. 

During the 3-month follow-up period, five out of nine deceased patients with malignant pathology died from septic or haemorrhagic shock after surgery and four from the progression of the primary disease. None of these patients suffered symptomatic VTE. Mortality in our cohort was higher than reported by Khorgami et al., who recorded five non-VTE-related deaths among 613 surgical patients, including general surgery patients [37], most probably due to different baseline characteristics of the patients. 

Infection with SARS-CoV-2 is a novel, important risk factor for VTE, also in association with recent surgery [5,6,38]. Recommendations for the prophylaxis of VTE in patients with cancer are similar in those with and without COVID [39]. Anticoagulant prophylaxis should be started between 12 h and 2 h preoperatively and continued for at least 7–10 days postoperatively with once-daily low-dose LMWH, preferably extended to 4 weeks after major cancer abdominal surgery in patients without a high bleeding risk [39]. Our practice conformed with these recommendations. Among 15 patients who had COVID-19 in the 3-month postoperative period, only one developed DVT. It was in the third month of follow-up after surgical treatment of an intraabdominal abscess, without a known malignancy. 

Postoperative haemorrhage is poorly predicted by existing assessment tools. However, it is generally recommended that pharmacological thromboprophylaxis be postponed in patients with a greater tendency to bleed; such patients may receive only mechanical thromboprophylaxis [16]. Generally, the bleeding rate from prophylactic doses of LWMH is low. In a systematic review of 33 trials on patients undergoing surgery and receiving pharmacological thromboprophylaxis, injection site bruising occurred in 6.9%, wound hematoma in 5.7%, drain site bleeding in 2.0%, and haematuria in 1.6%, while major bleeding from the gastrointestinal tract (0.2%) or retroperitoneal bleeding (<0.1%) was infrequent [40]. On the other hand, a recent meta-analysis found that the postoperative rate of clinically significant bleeding complications was 3.5% [41]. In our cohort, we detected five cases (2.2%) of non-fatal major bleeding, two of which could be attributed to LMWH. 

### Limitations

Our study has some important limitations. First, it was a single-centre, observational study with a heterogenous and relatively small sample size; hence, our results should be interpreted cautiously. Larger, well-designed, randomised controlled studies are needed to firmly validate our findings. Secondly, although the Caprini and Padua score groups did not differ in baseline characteristics, the patients were not randomized, and thus a small selection bias cannot be excluded. Our sample size was limited by the volume of the operated patients in the time-frame of the study. Since no power calculations of the sample size were performed, the absence of a statistically significant difference by no means implies equivalence or non-inferiority of either score. Thirdly, we only assessed for symptomatic VTE and did not perform systematic VTE screening, which might have contributed to the low incidence of non-fatal VTE. Asymptomatic VTE increases mortality among acutely ill medical patients [41]; therefore, we assessed death of any cause together with symptomatic VTE.

## 6. Conclusions

We found a relatively low incidence of mortality, non-fatal symptomatic VTE, and non-fatal major bleeding among elective abdominal surgery patients prescribed perioperative thromboprophylaxis based on either the Caprini or the Padua risk score, with no differences between the two groups. However, the correlation between the two risk scores was only moderate, and almost 40% of patients with a high Caprini score had a non-high Padua score, suggesting that the Caprini score may be better suited for VTE assessment in surgical patients than the Padua score. While all surgical patients should receive thromboprophylaxis based on risk assessment, we recommend an individualized approach in the decision process.

## Figures and Tables

**Table 1:** Baseline characteristics. The table includes also the number of confirmed COVID-19 cases in the 3-month observation period. The *p*-values for the comparisons of the two sub-groups are shown in the far-right column. (n—number of subjects, SD—standard deviation).

		All (n = 223)	Caprini Score Used (n = 122)	Padua Score Used (n = 101)	*p* (Caprini vs. Padua)
Gender	male, n (%)	118 (53)	59 (48)	59 (58)	0.134
	female, n (%)	105 (47)	63 (52)	43 (42)	
Age	years, mean (SD)	59.8 (14.7)	60.9 (14.9)	58.3 (14.1)	0.190
Diagnosis	non-malignant, n (%)	122 (55)	59 (48)	42 (42)	0.311
	malignant, n (%)	101 (45)	63 (52)	59 (58)	
Major surgery	n (%)	198 (89)	111 (92)	87 (86)	0.252
Smokers	n (%)	45 (20)	21 (20)	24 (24)	0.225
Confirmed COVID-19 during observation	n (%)	15 (6.7)	5 (4.1)	10 (9.9)	0.085

**Table 2:** The distribution of the Caprini score values and the Padua score values in relation to adverse outcomes.

		All (n = 223)	Caprini Score Used (n = 122)	Padua Score Used (n = 101)	*p* (Caprini vs. Padua Used)
Caprini score	Median	5	4.5	5	0.156
	25th percentile	4	4	4	
	75th percentile	7	7	7	
Padua score	Median	1	1	1	0.936
	25th percentile	0	0	0	
	75th percentile	4	3	4	
Adverse outcome	Composite	15	6	9	0.236
	Death	10	3	7	0.108
	VTE	5	3	2	0.810

**Table 3:** Adverse outcomes in relation to the VTE risk scores and baseline characteristics.

	Composite Adverse Outcome		Death		Symptomatic Venous Thromboembolism	
	With/All (No.)	OR (95% CI), *p*	With/All (No.)	OR (95% CI), *p*	With/All (No.)	OR (95% CI), *p*
High vs. Non-high Caprini Score	15/151 vs. 0/72	∞ (∞), -	10/151 vs. 0/72	∞ (∞), -	5/151 vs. 0/72	∞ (∞), -
High vs. Non-high Padua Score	10/67 vs. 5/156	5.30 (1.74–16.17), *p* = 0.0017	9/67 vs. 1/156	24.05 (2.98–194.05), *p* = 0.0014	1/67 vs. 4/156	0.58 (0.06–5.25), *p* = 0.3122
Malignant vs. Benign Diagnosis	10/101 vs. 5/122	2.57 (0.85–7.79), *p* = 0.0473	9/101 vs. 1/122	11.84 (1.47–95.1), *p* = 0.0100	1/101 vs. 4/122	0.30 (0.003–2.68), *p* = 0.1392
Male vs. Female Gender	9/118 vs. 6/105	1.36 (0.47–3.96), *p* = 0.2852	5/118 vs. 5/105	0.88 (0.25–3.15), *p* = 0.4251	4/118 vs. 1/105	3.65 (0.40–33.18), *p* = 0.1251
Age ≥65 y vs. <65 y	12/96 vs. 3/127	5.90 (1.62–21.56), *p* = 0.0036	7/96 vs. 3/127	3.25 (0.82–12.92), *p* = 0.0469	5/96 vs. 0/127	∞ (∞), -
Smoker vs. Non-smoker	6/45 vs. 9/178	2.89 (0.97–8.59), *p* = 0.0282	4/45 vs. 6/178	2.80 (0.75–10.37), *p* = 0.0619	2/45 vs. 3/178	2.71 (0.44–16.75), *p* = 0.1412
COVID-19 vs. No COVID-19	2/15 vs. 13/208	2.31 (0.47–11.33), *p* = 0.1514	1/15 vs. 9/208	1.58 (0.19–13.37), *p* = 0.3374	1/15 vs. 4/208	3.64 (0.38–34.81), *p* = 0.1308

## Data Availability

The data presented in this study are available on request from the corresponding author. The data are not publicly available due to patients’ privacy.

## Supplementary Materials

The following supporting information can be downloaded at: https://www.mdpi.com/article/10.3390/life12111843/s1, Table S1: Benign diagnoses as reasons for abdominal surgery in our cohort; Table S2: Malignant diagnoses as reasons for abdominal surgery in our cohort; Table S3: Original Caprini score questionnaire (COPD—chronic obstructive pulmonary disease, BMI—body mass index, SVT—superficial venous thrombosis, DVT—deep venous thrombosis, PE—pulmonary embolism) [17]; Table S4: The Padua score questionnaire (BMI—body mass index) [18].
