# Supplementary material for: Perioperative Prevention of Venous Thromboembolism in Abdominal Surgery Patients Based on the Caprini or the Padua Risk Score—A Single Centre Prospective Observational Study
Source: Life (Basel). 2022 Nov 11;12(11):1843. doi: 10.3390/life12111843 (PMC9694484; doi:10.3390/life12111843)
Supplement: Supplementary file 1 [file life-12-01843-s001.zip › life-1969357-supplementary.pdf]

Table S1. Benign diagnoses as reasons for abdominal surgery in our cohort.

Benign diagnosis/Nr of patients	Total: 121
Cholecystholithiasis	43 (35,5%)
Polyp of the gallbladder	3 (2,5%)
Hernias (inguinal, postoperative and umbilical)	41 (33,9%)
Occlusion of stomata	6 (5,0%)
Stenosis of ileum because of IBD	2 (1,7%)
Perianal fistula	3 (2,5%)
Other	25 (20,7%)

Table S2. Malignant diagnoses as reasons for abdominal surgery in our cohort.

Malignant diagnosis/Nr of patients	Total: 96
Adenocarcinoma of colon	27 (28,1%)
Adenocarcinoma of rectum	4 (4,2%)
Adenocarcinoma or NET of pancreas	26 (27,1%)
Adenocarcinoma, GIST or NET of stomach	20 (20,1%)
Adenocarcinoma of gallbladder	3 (3,1%)
Liver metastasis of colonic adenocarcinoma	5 (5,2%)
Hepatocellular carcinoma	3 (3,1%)
Cholangiocarcinoma	4 (4,2%)
Other	4 (4,2%)

Table S3. Original Caprini score questionnaire (COPD – chronic obstructive pulmonary disease, BMI – body mass index, SVT – superficial venous thrombosis, DVT- deep venous thrombosis, PE – pulmonary embolism) [Caprini JA. Thrombosis risk assessment as a guide to quality patient care. Dis Mon. 2005 Feb-Mar;51(2-3):70-8. doi: 10.1016/j.disamonth.2005.02.003.]

Risk factors adding 1 point each:
Age 41-60 years.
Minor surgery planned.
History of prior major surgery.
Varicose veins.
History of inflammatory bowel disease.
Swollen legs (current).
Obesity (BMI >30).
Acute myocardial infarction (<1 month).
Congestive heart failure (<1 month).
Sepsis (<1 month).
Serious lung disease incl.
Pneumonia (<1 month).
Abnormal pulmonary function (COPD).
Medical patient currently at bed rest.
Leg plaster cast or brace.
Other risk factors.

Risk factors adding 2 points each:
Age 60-74 years.
Major surgery (>60 minutes).
Arthroscopic surgery (>60 minutes).
Laparoscopic surgery (>60 minutes).
Previous malignancy.
Central venous access.

Risk factors adding 3 points each:
Age over 75 years.
Major surgery lasting 2-3 hours.
BMI >50 (venous stasis syndrome).
History of SVT, DVT/PE.
Family history of DVT/PE.
Present cancer or chemotherapy.
Positive Factor V Leiden.
Positive Prothrombin 20210A.
Elevated serum homocysteine.
Positive Lupus anticoagulant.
Elevated anticardiolipin antibodies.
Heparin-induced thrombocytopenia.

Risk factors adding 5 points each:
Elective major lower extremity arthroplasty.
Hip, pelvis or leg fracture (<1 month).
Stroke (<1 month).
Multiple trauma (<1 month).
Acute spinal cord injury (paralysis) (<1 month).
Major surgery lasting over 3 hours.

Risk factors for women, adding 1 point each:
Oral contraceptives or hormone replacement therapy.
Pregnancy or postpartum (<1 month).
History of unexplained stillborn infant, recurrent spontaneous abortion (≥ 3), premature birth with toxemia or growth-restricted infant.

Table S4. The Padua score questionnaire (BMI- body mass index) [Barbar S, Noventa F, Rossetto V, Ferrari A, Brandolin B, Perlati M, De Bon E, Tormene D, Pagnan A, Prandoni P. A risk assessment model for the identification of hospitalized medical patients at risk for venous thromboembolism: the Padua Prediction Score. J Thromb Haemost. 2010 Nov; 8(11):2450-7. doi: 10.1111/j.1538-7836.2010.04044.x.

**Risk factors and corresponding points:**	
Active cancer	3
Previous VTE (with the exclusion of superficial vein thrombosis)	3
Reduced mobility	3
Already known thrombophilic condition	3
Recent (≤1 month) trauma and/or surgery	2
Elderly age (≥70 years)	1
Heart and/or respiratory failure	1
Acute myocardial infarction or ischemic stroke	1
Acute infection and/or rheumatologic disorder	1
Obesity (BMI ≥30	1
Ongoing hormonal treatment	1
